# Supplementary material for: Development of visual response selectivity in cortical GABAergic interneurons
Source: Nat Commun. 2022 Jul 1;13:3791. doi: 10.1038/s41467-022-31284-6 (PMC9249896; doi:10.1038/s41467-022-31284-6)
Supplement: Supplementary file 1 — Supplementary Information [file 41467_2022_31284_MOESM1_ESM.pdf]

Supplementary Information for

Development of visual response selectivity in cortical GABAergic interneurons

Jeremy T. Chang* and David Fitzpatrick

*Correspondence should be addressed to Jeremy Chang (jeremy.chang@mpfi.org)

This PDF file includes:

- Supplementary Figure 1-5

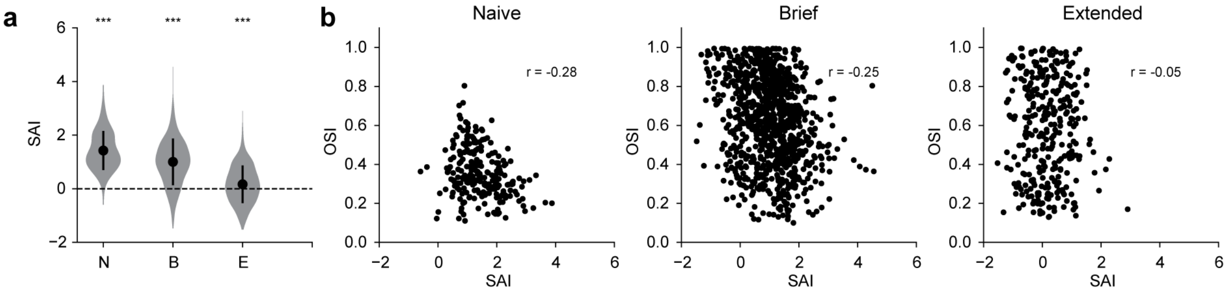

Supplementary Fig. 1 Visual responsiveness to the non-preferred orientation contributes to weak orientation selectivity early in development. (a) Violin plots and Mean ± SD of Stimulus Activity Index (SAI) of significantly orientation selective GABA-INs for Naive (N, $p<0.0001$, n=233 cells, 7 animals), Brief (B, $p<0.0001$, n = 966 cells, 6 animals), and Extended (E, $p<0.0001$, n =326 cells, 5 animals) experience. ***: $p < 0.005$, Two-tailed Student's t-test. (b) Scatter plots of the discriminability of the orthogonal orientation and blank trials (Orthogonal-Blank Cohen's *d*) versus orientation selectivity index (OSI) for Naive (left), Brief (middle) and Extended (right) experience. Cells shown were significantly selective for orientation. (Naive r = -0.28, p <0.0001, n=233 cells, 7 animals; Brief r = -0.25, p<0.0001, n=966 cells, 6 animals; Extended r= -0.05, p=0.3730, n=326 cells, 5 animals; Pearson's r). Source data are provided as a Source Data file.

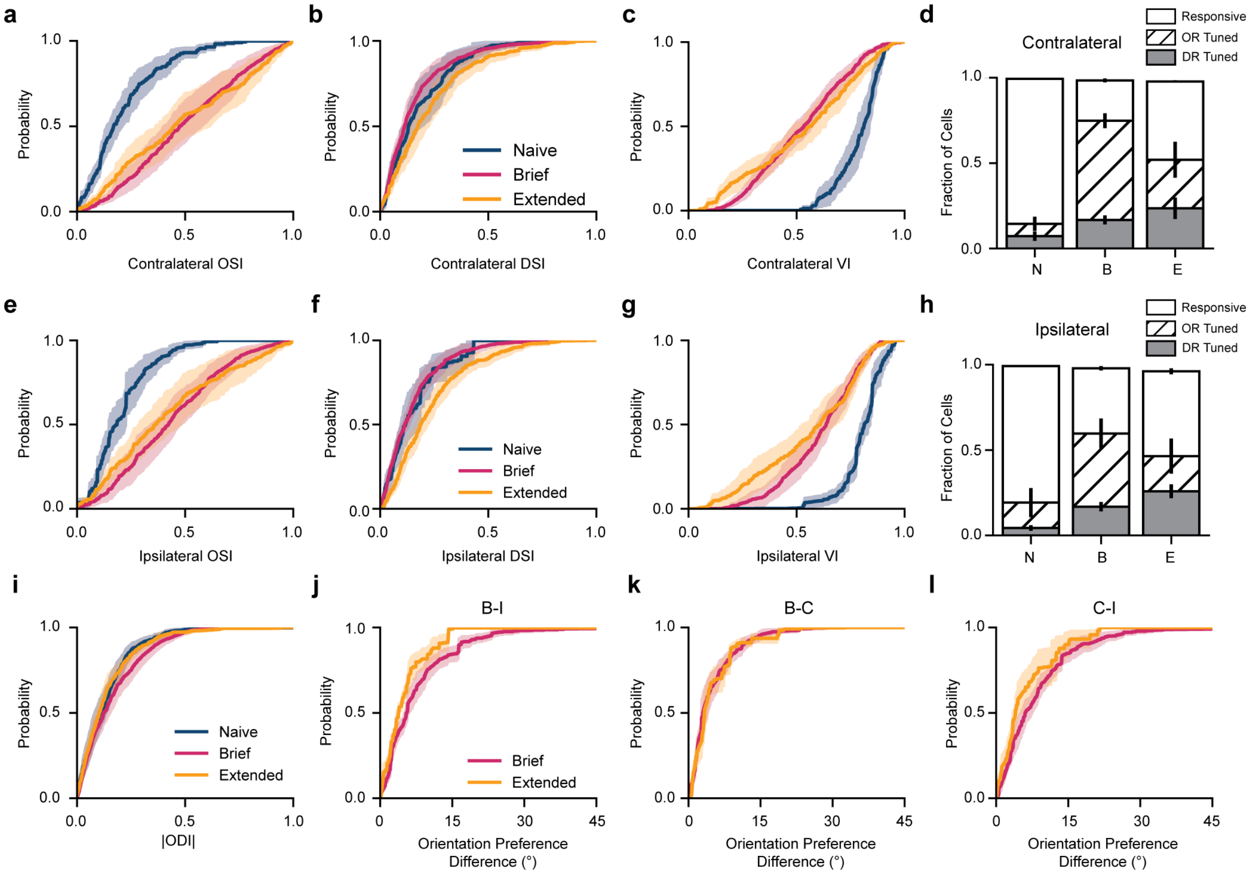

Supplementary Fig. 2 Binocular orientation binocular preferences develop in an aligned manner. (a-c) Cumulative plots for orientation selectivity index (a), direction selectivity index (b), and variability index (c) for contralateral stimulus presentation for Naive (blue), Brief (Magenta), and Extended (gold) experience. Mean ± SEM. (d) Fractions of cells that are responsive (open), significantly orientation tuned (hatched), or significantly direction tuned (gray) for Naive (N, n=7), Brief experience (B, n=6), and Extended (E) experience. Error bars denote SEM. (e-g) Same as A-B but for ipsilateral stimulus presentation. (h) Same as D but for ipsilateral stimulus presentation. (i) Cumulative plot of monocularity (|ODI|) for Naive (blue, n=7), Brief (magenta, n=6), and Extended (gold, n=6) experience. (j) Cumulative plot of the difference in preferred orientation for binocular versus ipsilateral stimulus presentation. (k) Cumulative plot of the difference in preferred orientation for binocular versus contralateral stimulus presentation. (l) Cumulative plot of the difference in preferred orientation for contralateral versus ipsilateral stimulus presentation. Mean ± SEM. For all plots Naive (n=7), Brief (n=6), and Extended (n=7). Source data are provided as a Source Data file.

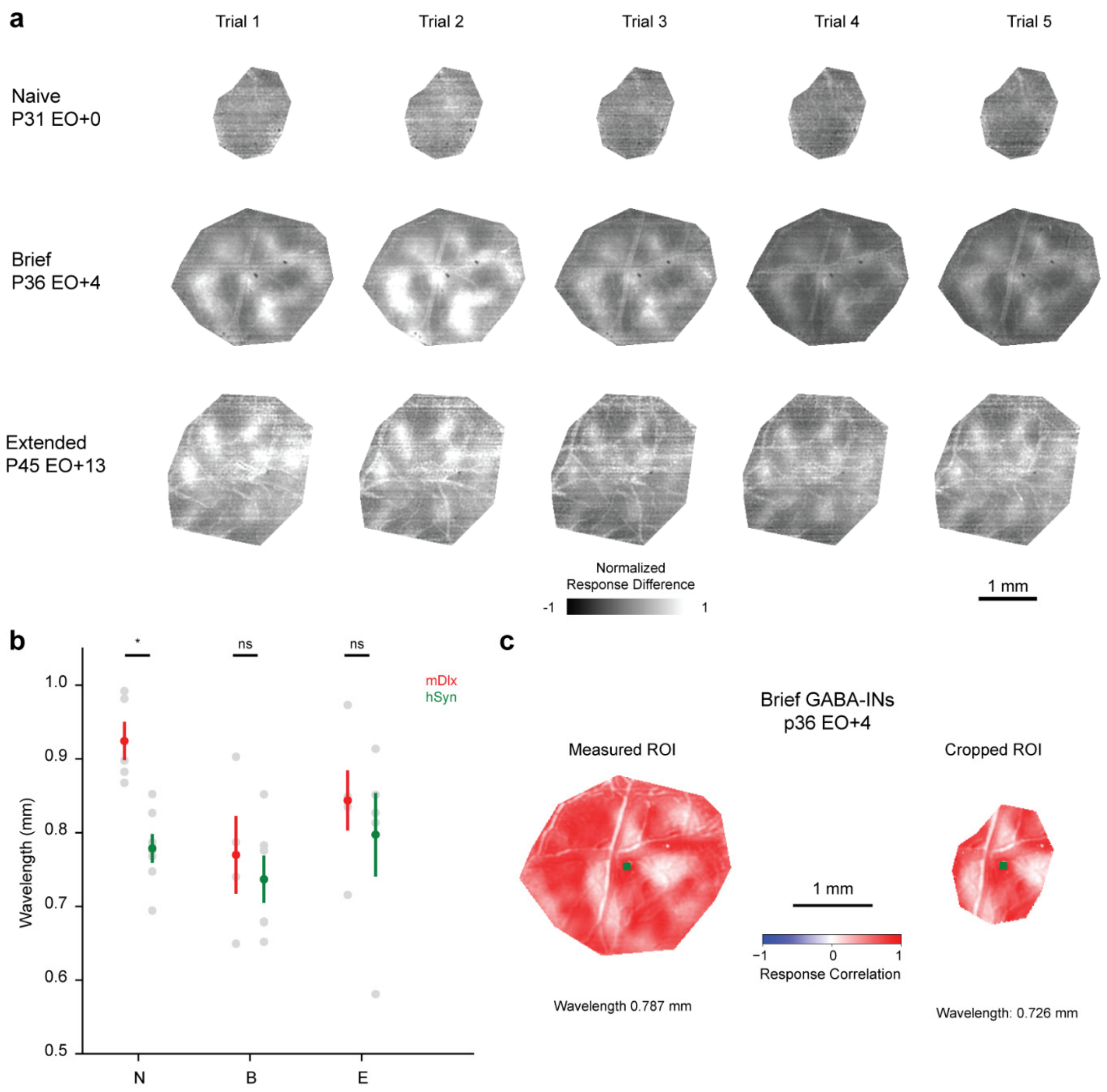

Supplementary Fig. 3 Widefield Single trial responses become more reliable with visual experience. (a) Example widefield epifluorescence responses for five trials of a single stimulus presentation for example Naive, Brief, and Extended experience animals. (b) Characteristic wavelength of responses for GABA-INs (mDlx, red) and excitatory neurons (hSyn, green) for response correlation maps. Mean +/- SEM. Grey dots denote individual animals. Two-tailed bootstrap test *: $p<0.05$, ns: not significant. Naive ($p=0.0048$, n= 7 mDlx vs 5 hSyn), Brief ($p=0.5120$, n=6 mDlx vs 4 hSyn), Extended ($p=0.4730$, n= 5 mDlx vs 5 hSyn). (c) Example full pairwise pixel correlation map for a Brief experience animal (left, reproduced from Fig. 2c). Cropped pairwise pixel correlation map for a Brief animal with modular activity (right). The region of interest is the same size as the one for the Naive animal shown in Fig. 2c. Even with the smaller field of view a comparable wavelength was computed using the wavelet fitting column spacing method. Source data are provided as a Source Data file.

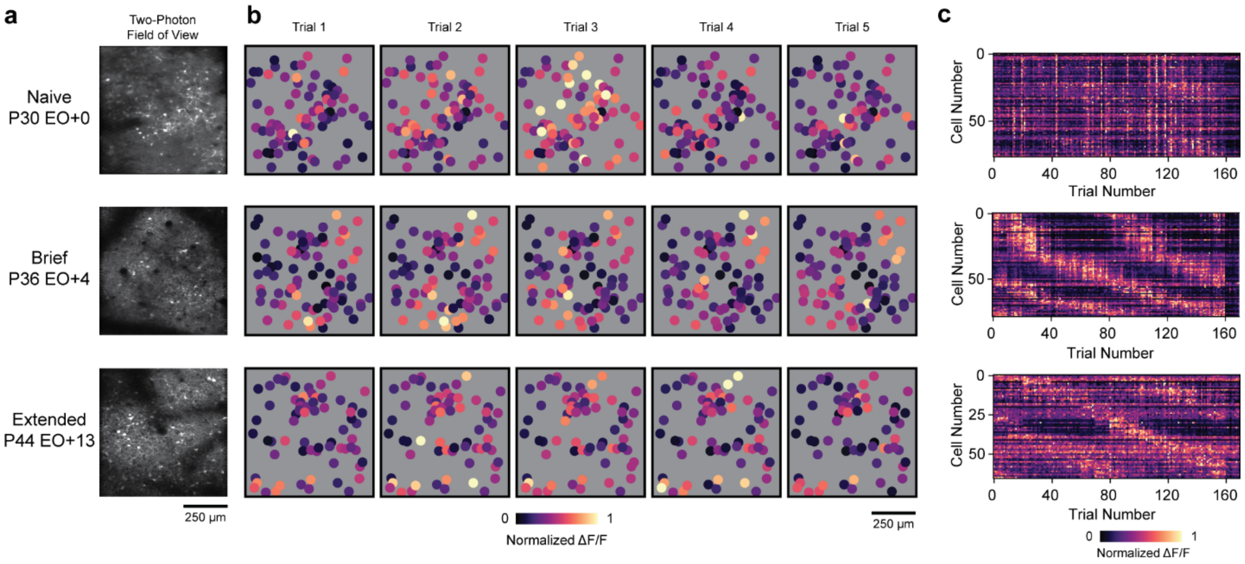

Supplementary Fig. 4 Cellular Single trial responses become more reliable with visual experience. (a) Example two-photon fields of view for example Naive, Brief, and Extended experience animal. (b) Examples of normalized population responses for five trials of a single stimulus presentation for the corresponding animals shown in (a). (c) Raster plots of trial responses for the corresponding animals shown in (a). Responses were normalized per cell to the peak response. Cells are sorted by preferred direction. Stimuli were sorted by direction such that each group of 10 trials corresponds to a single stimulus direction. The last 10 trials are blank trials to show spontaneous activity.

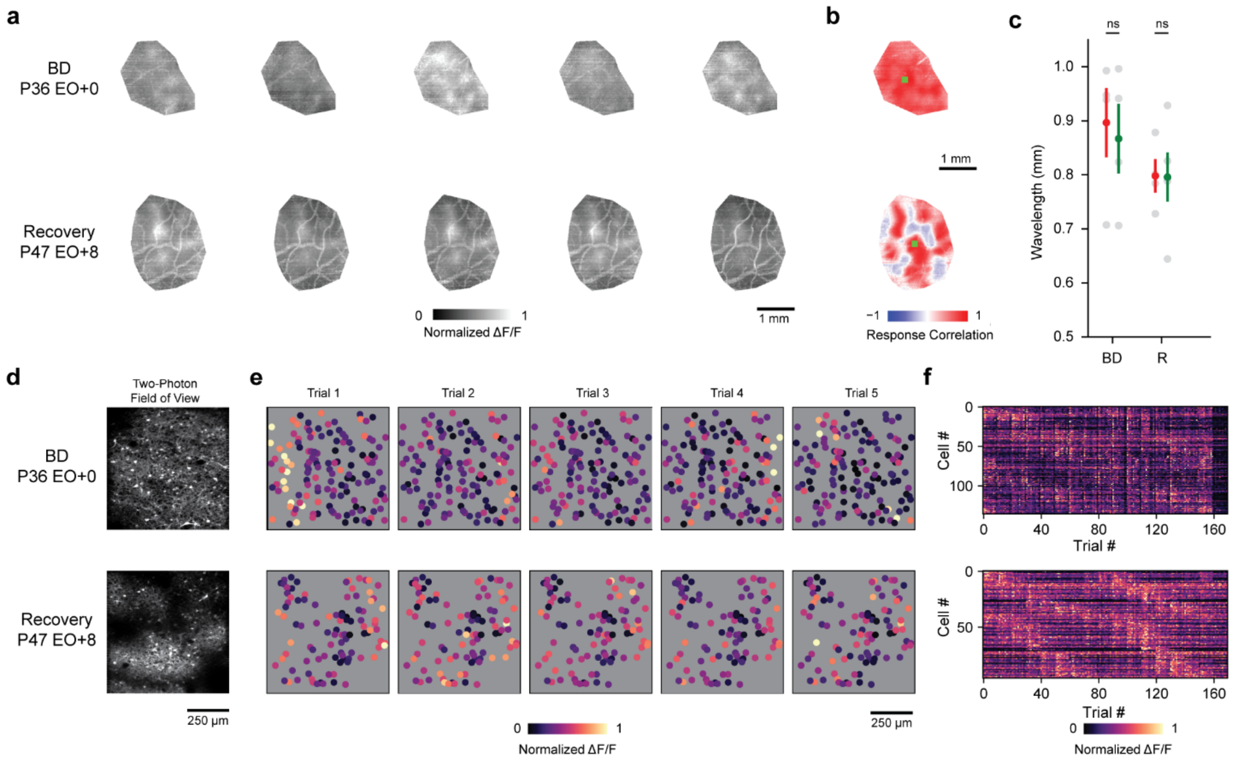

Supplementary Fig. 5 Delayed visual experience is sufficient to develop reliable responses. (a) Example widefield epifluorescence responses for five trials of a single stimulus presentation for example Binocular Deprivation and Recovery animals. (b) Example pairwise pixel response correlation maps for example Binocular Deprivation and Recovery animals. Green squares identify the seed positions. (c) Characteristic wavelength of responses for GABA-INs (mDlx, red) and excitatory neurons (hSyn, green) for response correlation maps. Mean ± SEM. Grey dots denote individual animals. Two-tailed bootstrap test, ns: not significant. For mDlx BD (n=4) and (n=4). For hSyn BD (n=4) and Recovery (n=5). (d) Example two-photon fields of view for a Binocular Deprivation and Recovery animal. (e) Examples of normalized population responses for five trials of a single stimulus presentation for the corresponding animals shown in (d). Examples are representative for both BD (n=4) and Recovery (n=5) measurements. (f) Raster plots of trial responses for the corresponding animals shown in (c). Responses were normalized per cell to the peak response. Cells are sorted by preferred direction. Stimuli were sorted by direction such that each group of 10 trials corresponds to a single stimulus direction. The last 10 trials are blank trials to show spontaneous activity. Source data are provided as a Source Data file.
