# Supplementary material for: Development of visual response selectivity in cortical GABAergic interneurons
Source: Nat Commun. 2022 Jul 1;13:3791. doi: 10.1038/s41467-022-31284-6 (PMC9249896; doi:10.1038/s41467-022-31284-6)
Supplement: Supplementary file 3 — Reporting Summary [file 41467_2022_31284_MOESM3_ESM.pdf]

nature portfolio

Corresponding author(s): Jeremy Chang
Last updated by author(s): Apr 22, 2022

Reporting Summary

Nature Portfolio wishes to improve the reproducibility of the work that we publish. This form provides structure for consistency and transparency in reporting. For further information on Nature Portfolio policies, see our Editorial Policies and the Editorial Policy Checklist.

Statistics

For all statistical analyses, confirm that the following items are present in the figure legend, table legend, main text, or Methods section.

n/a	Confirmed	
[ ]	[x]	The exact sample size (*n*) for each experimental group/condition, given as a discrete number and unit of measurement
[ ]	[x]	A statement on whether measurements were taken from distinct samples or whether the same sample was measured repeatedly
[ ]	[x]	The statistical test(s) used AND whether they are one- or two-sided<br>*Only common tests should be described solely by name; describe more complex techniques in the Methods section.*
[x]	[ ]	A description of all covariates tested
[ ]	[x]	A description of any assumptions or corrections, such as tests of normality and adjustment for multiple comparisons
[ ]	[x]	A full description of the statistical parameters including central tendency (e.g. means) or other basic estimates (e.g. regression coefficient) AND variation (e.g. standard deviation) or associated estimates of uncertainty (e.g. confidence intervals)
[ ]	[x]	For null hypothesis testing, the test statistic (e.g. *F*, *t*, *r*) with confidence intervals, effect sizes, degrees of freedom and *P* value noted<br>*Give P values as exact values whenever suitable.*
[x]	[ ]	For Bayesian analysis, information on the choice of priors and Markov chain Monte Carlo settings
[x]	[ ]	For hierarchical and complex designs, identification of the appropriate level for tests and full reporting of outcomes
[ ]	[x]	Estimates of effect sizes (e.g. Cohen's *d*, Pearson's *r*), indicating how they were calculated

Our web collection on statistics for biologists contains articles on many of the points above.

Software and code

Policy information about availability of computer code

Data collection	The following commercial or open source software were used: µManager v1.4.16; ScanImage version 2015a, MATLAB versions 2016a and 2018b; Psychopy v1.85; Fiji v1.51n; Cell Magic Wand version 1.0, Spike 2 v7.11b
Data analysis	ROIs for widefield imaging data was identified using Fiji (v1.51n). Two-photon ROIs were identified using Fiji (v1.51n) and the CellMagicWand plugin (https://github.com/fitzlab/CellMagicWand). All data generated were analyzed using Matlab (2016a & 2018b) or Python 3.7 with the following plugins: PIMS v0.4.1, imageio v2.4.1, Numpy v1.15.1, read-roi v1.4.2, scikit-image v0.14.0, scikit-learn v1.2.1, scipy v1.1.0, and pandas v0.23.4. Code for data analysis can be made available upon reasonable request to the corresponding author.

For manuscripts utilizing custom algorithms or software that are central to the research but not yet described in published literature, software must be made available to editors and reviewers. We strongly encourage code deposition in a community repository (e.g. GitHub). See the Nature Portfolio guidelines for submitting code & software for further information.

Data

Policy information about availability of data

All manuscripts must include a data availability statement. This statement should provide the following information, where applicable:

- Accession codes, unique identifiers, or web links for publicly available datasets
- A description of any restrictions on data availability
- For clinical datasets or third party data, please ensure that the statement adheres to our policy

Data availability statement is included in our methods section. Source data are provided for the following figures: Fig 1f-l; 2c,e; 3d,e,g; 4e-h; 5b-d,f; Supplementary Fig. 1a-b; 2a-l; 3b; 5c. All data are available upon reasonable request from the corresponding author.

Field-specific reporting

Please select the one below that is the best fit for your research. If you are not sure, read the appropriate sections before making your selection.

☒ Life sciences ☐ Behavioural & social sciences ☐ Ecological, evolutionary & environmental sciences

For a reference copy of the document with all sections, see nature.com/documents/nr-reporting-summary-flat.pdf

Life sciences study design

All studies must disclose on these points even when the disclosure is negative.

Sample size	No a priori sample size calculations were performed. 3+ animals is an accepted sample size for in vivo imaging in carnivores such as ferrets and cats (see for example Wilson et al. 2016., Chang et al. 2020, Mulholland et al. 2021).
Data exclusions	Imaging data was only excluded from animals where GCaMP6s expression was unacceptably low.
Replication	All attempts at replication were successful, and all imaged animals are reflected in the data. Our observations of orientation selectivity of GABA-INs in animals with visual experience were consistent with previous studies (Wilson et al. 2016).
Randomization	All comparisons were made within experimental group (e.g. animals with closed eyelids before eye-opening, animals imaged with 4-7 days of experience, and animals with more than 8 days of experience), and animals were allocated to an experimental group sequentially.
Blinding	No attempts at blinding were performed in this study, as it it not possible to remain blind to animal age or eye opening status.

Reporting for specific materials, systems and methods

We require information from authors about some types of materials, experimental systems and methods used in many studies. Here, indicate whether each material, system or method listed is relevant to your study. If you are not sure if a list item applies to your research, read the appropriate section before selecting a response.

Materials & experimental systems

n/a	Involved in the study	
☒	☐	Antibodies
☒	☐	Eukaryotic cell lines
☒	☐	Palaeontology and archaeology
☐	☒	Animals and other organisms
☒	☐	Human research participants
☒	☐	Clinical data
☒	☐	Dual use research of concern

Methods

n/a	Involved in the study	
☒	☐	ChIP-seq
☒	☐	Flow cytometry
☒	☐	MRI-based neuroimaging

Animals and other organisms

Policy information about studies involving animals; ARRIVE guidelines recommended for reporting animal research

Laboratory animals	Female ferrets (Marshall Farms, P27-55)
Wild animals	No wild animals were used.
Field-collected samples	No field-collected samples were used
Ethics oversight	All experimental procedures were approved by the Max Planck Florida Institute for Neuroscience Institutional Animal Care and use committee and were performed in accordance with guidelines from the U.S. National Institute of Health.

Note that full information on the approval of the study protocol must also be provided in the manuscript.
